# Knowledge and barriers among Lebanese community pharmacists in pediatric antibiotic dosing: a cross-sectional study

**DOI:** 10.3389/fped.2025.1630682

**Published:** 2025-09-01

**Authors:** Stephanie Akiki, Joyce Saab, Jihan Safwan, Bahia Chahine, Mohamad Rahal

**Affiliations:** ^1^ School of Pharmacy, Lebanese International University, Beirut, Lebanon; ^2^ INSPECT-LB (Institut National de Santé Publique, d’Épidémiologie Clinique et de Toxicologie-Liban), Beirut, Lebanon

**Keywords:** antibiotics resistance, community pharmacists, pharmacist education, dosing errors, health knowledge, public health Lebanon, pediatrics

## Abstract

**Background:**

The accurate dosing of antibiotics in pediatric patients is critically important in Lebanon due to the high prevalence of antibiotic misuse, widespread over-the-counter access without prescriptions, and limited healthcare access for a significant portion of the population. The study aimed to assess the knowledge of Lebanese community pharmacists regarding proper antibiotic dosing in pediatric patients, identify the factors that influence their knowledge, and explore the barriers that lead to improper dosing of antibiotics.

**Methods:**

A cross-sectional questionnaire-based study was conducted where 309 community pharmacists were recruited through social media platforms to complete a self-administered questionnaire. The survey consisted of seven sections, including demographic characteristics, case scenarios, and barriers related questions about pediatric antibiotic dosing. Chi-square test assessed associations; variables with *p* ≤ 0.2 were included in the logistic regression. Significance was set at *P* ≤ 0.05.

**Results:**

84.1% of community pharmacists had adequate knowledge about antibiotics dosing in pediatric patients. Monthly income exceeding 5,000,001 Lebanese Pounds (LBP) (CI: 3.135–12.434, *P* = 0) and working more than 8 h per day (CI: 0.112–0.938, *P* = 0.038) were significantly associated with knowledge levels. Pharmacists were mostly knowledgeable regarding the dosing of amoxicillin-clavulanate in acute otitis media with the highest correct response rate (98%), while the case of trimethoprim-sulfamethoxazole dosing in urinary tract infection had the lowest correct response rate (75.1%). Lack of scientific knowledge was reported as the most common barrier causing dosing errors.

**Conclusions:**

The study concluded that most community pharmacists have appropriate knowledge about the correct antibiotic dosage for pediatric patients. However, there are still barriers that need to be addressed to improve pharmacists' practice and reduce dosing errors which can lead to serious consequences such as therapeutic failure, antibiotic resistance, toxicity and side effects, altered gut microbiota, increased healthcare burden, and negative impacts on a child's growth and development. The authorities should consider implementing more effective platforms and targeted educational programs to keep pharmacists informed and up-to-date, which could enhance the healthcare sector.

## 1 Background

Antibiotics are one of the most effective weapons we have in the fight against life-threatening infections; it is vital to emphasize how many lives they have saved or how much they have contributed to the control of infectious diseases (1). Misuse of these drugs, can be extremely dangerous and can manifest itself in a variety of ways: using the wrong antibiotic, incorrect antibiotic formulation, inappropriate dosage and inappropriate duration (2).

Globally, sepsis and antimicrobial resistance (AMR) caused a major health burden in 2021, with sepsis responsible for 21.4 million deaths, including 2.68 million children under five, and antimicrobial resistance (AMR) contributing to up to 4.71 million deaths, 1.14 million of which were directly due to resistant infections (3).

Although national data on sepsis and AMR-related mortality in Lebanon is lacking, hospital-based studies reveal high fatality rates from drug-resistant infections, with mortality reaching up to 33% for Acinetobacter baumannii and 25.6% for Carbapenem-Resistant Enterobacterales (CRE) (4).

Antibiotics are the most commonly prescribed medications in the pediatric population, both nationally and internationally, with a prevalence of 47.3%, and almost half of all prescriptions are unnecessary (5). Children are not little adults; the adoption of a one-dose-fits-all method for prescribing medications in children is impossible due to age-related changes in drug absorption, distribution, metabolism, and elimination (6, 7). For this reason, dosing errors are the most common type of drug error among pediatrics, because pediatric patients are a special population that needs particular attention necessitating multiple dose calculations based on various factors like the patient's age, weight and clinical state (8, 9).

The abuse of antibiotics in children is a serious problem that has received little attention in epidemiological studies and antibiotics resistance constitutes a significant issue, described by the WHO as “a global public health concern,” particularly affecting children (10). It could be caused by a variety of circumstances, including the medication itself, the treating doctor, or even the pharmacist (11). In the United States, a significant proportion of community-prescribed antibiotics is administered to children, with studies indicating that nearly 50% of these prescriptions are unnecessary. In Canada, an estimated 28% of antibiotic prescriptions for children aged 2–18 years and 24% for those under 2 years were deemed unnecessary, indicating a high prevalence of improper antibiotic use in pediatric populations (12, 13).

Moving to the Middle Eastern region, similar concerns regarding inappropriate antibiotic use are evident. Keewan et al. showed that most of the pharmacists in Jordan were non-knowledgeable about the appropriate dosing of antibiotics in pediatrics (14). Their monthly income significantly affected their knowledge, poor scientific knowledge about the correct dose calculation was the most leading cause of these results, and implementing appropriate educational programs and guidelines to regulate antibiotics practice among community pharmacists are highly recommended (14).

Locally, in a recent Lebanese study, 84.6% of pharmacists prescribed antibiotics for children in community pharmacies; and the majority of pharmacists claimed parental pressure as a cause for prescribing antibiotics. Furthermore, Hallit et al. reported that there is a proportion of antibiotics that were inappropriately dispensed in terms of dose and duration of treatment among Lebanese community pharmacists (15). Additionally, in Zahreddine et al. study, Lebanese pharmacists blamed first parents, second physicians, and third themselves for antibiotic overuse (11). Poor knowledge was found in pharmacists with a higher number of years of experience; which is comparable to the results of a Saudi Arabian study, showing that pharmacists with a job experience ranging between three to four years had better knowledge towards the appropriate use of drugs compared to those with a nine to ten-year experience (16). Both study results demonstrated poor practices of community pharmacists towards dispensing antibiotics making continuous education a necessity to update and improve the pharmacists' knowledge about antibiotics use in pediatrics.

Community pharmacies are the primary source of antibiotics in Lebanon, and pharmacists are widely regarded as the first port of call for healthcare advice and services due to their convenient accessibility, especially for the pediatric population during the socio-economic crisis in the country where most of the parents are not being able to finance a doctor's visit anymore. Limited access to medical care in Lebanon, driven by a collapsed healthcare system, soaring out-of-pocket costs, widespread shortages of medications, and the emigration of healthcare workers, has left many households unable to access basic health services (17). While specific data on the percentage of pediatric antibiotics dispensed without a prescription in Lebanon is limited, available studies found that approximately 68.7% of pharmacies dispensed antibiotics without prescriptions, indicating a significant level of over-the-counter antibiotic use in Lebanon (18). Thus, pharmacists are crucial players in the process of treating those populations (15). Having the essential knowledge is key in preventing prescribing errors and poor medical knowledge might be a leading cause to it. Given the lack of recent data on pharmacists' knowledge of antibiotic dosing in Lebanon, this study was conducted to evaluate the knowledge of community pharmacists in Lebanon regarding the appropriate dosing of antibiotics among pediatrics, and to identify other barriers, such as parental pressure vs. knowledge gaps, to inappropriate antibiotics dosing and the possible consequences of these errors.

## 2 Methods

### 2.1 Study design

A cross-sectional study involving Lebanese community pharmacists was conducted between February and June 2022. A snowball sampling technique was used to gather information on pharmacists from different Lebanese regions (Beirut, Mount Lebanon, North, Akkar, South, Bekaa and Baalbek/Hermel). This technique may introduce selection bias due to the non-random nature of participant referral. Initial contacts were identified through a combination of professional networks, and existing relationships with community pharmacists. No professional associations were involved. A self-administered online survey was used to assess the knowledge of Lebanese community pharmacists about the appropriate dosing of antibiotics among pediatric patients, and was sent via an electronic link. The survey was adapted from a validated questionnaire developed by Keewan et al.

### 2.2 Selection and recruitment of the study participants

The study was conducted only on the Lebanese community pharmacists. Other nationalities or participants other than community pharmacists were excluded from the study.

### 2.3 Data collection

The online survey was drafted in English, a language that is comprehended by all pharmacists in Lebanon, and it was piloted with 10 pharmacists to ensure that the content and clarity were appropriate. The finalized questionnaire was subsequently distributed along with its link to all community pharmacists practicing in Lebanon after the necessary modifications had been made. The survey was done in accordance with the applicable data protection law, and the survey's scope and purpose were made clear at the beginning of the questionnaire, confirming its strict confidentiality. The study's participants were made aware that taking part was completely voluntary, and they received assurance that their answers would be treated confidentially and anonymously. Before starting the survey, they were asked to check an obligatory option box to say that this was their first time participating (ensuring a 100% consent rate).

### 2.4 Ethical approval

The study protocol, which adhered to the Declaration of Helsinki Ethical Guidelines for Medical Research Involving Human Subjects, was approved by the ethics and research committee of the Lebanese International University School of Pharmacy (protocol number: LIUSOP-RC-033). The process pertaining to the confidentiality of the data and information given to the volunteers were foreseen, and the nature of the elements to be collected did not pose the risk of revealing weaknesses unknown to the volunteer and so producing unforeseen reactions.

### 2.5 Sample size calculation

The total number of registered community pharmacists was obtained from the website of the OPL (Order of Pharmacists of Lebanon). Epi-info software was used to calculate the sample size of the current study. Based on a similar Jordanian study, it was assumed that 13.6% of the Lebanese community pharmacists had sufficient knowledge about antibiotics dosing errors in pediatrics. Using an absolute precision of 5%, the sample size was computed at a 95% confidence interval. In order to obtain a representative sample for the current study, a sample size of 172 was calculated. The required sample size was increased to allow for subjects lost during the study and incomplete responses. Accordingly, the estimated sample size was 309.

### 2.6 Questionnaire development and structure

Due to restrictions during the COVID 19 (*coronavirus disease of 2019)* pandemic in Lebanon, the data was collected using a google form questionnaire which was prepared by the research team based on literature review, and was sent out randomly via WhatsApp Messenger and other social media applications to the Lebanese community pharmacists. The survey consisted of seven sections covering all main key points of the research topic. The first two sections included a small introduction to the questionnaire outlining the scope, purpose of the study and anonymity of personal participant information, followed by an informed consent statement. Socio-demographic characteristics were present in the third section. To assess the community pharmacists' knowledge about the appropriate dosing of the most commonly encountered antibiotics in pediatric patients, 4 case scenarios about common pediatric infections were included in the fourth section. The last three sections comprised 5-point Likert scale questions to evaluate the pharmacists' practice and perception regarding antibiotics dosing errors in pediatrics. The content of the survey was checked for validity with the assistance of an epidemiologist.

### 2.7 Knowledge score

A knowledge score was created to evaluate the participants' expertise about appropriate antibiotic dosing in pediatrics. The score ranged from 0 to 4, with one point assigned for each accurate response and zero for each incorrect or unclear response. Further, the participant's degree of knowledge was divided into two categories depending on the number of correct cases, where a high level of knowledge was associated with more than two correct cases and low level of knowledge was related to 2 or fewer correct cases (14).

### 2.8 Statistical analysis

The respondents' demographic traits were evaluated using descriptive statistical analysis. For categorical data, frequencies and percentages were utilized, and median (±interquartile range) was used for continuous variables. When a normal distribution was assumed, the student (independent) *T*-test was employed to evaluate whether there was a relationship between the qualitative and quantitative variables. To determine the association between two qualitative variables Chi-square test was used. A *P*-value of ≤0.05 was deemed statistically significant.

The bivariate analyses' significant predictors with *p*-values ≤ 0.2 were then exported to the multivariate logistic regression model to identify independent predictors. Multicollinearity among independent variables was assessed using Variance Inflation Factors (VIF), and no concerning multicollinearity was detected (VIF < 10). Model fit was evaluated using the Hosmer-Lemeshow goodness-of-fit test, which showed an adequate fit. An alpha of 0.05 was used to determine statistical significance. All analyses were performed using the IBM's Statistical Package for the Social Sciences (SPSS) version 26 (IBM, Inc, Chicago, IL).

## 3 Results

### 3.1 Socio-demographic characteristics and details related to antibiotics dispensing

A total of 309 participants were included from all Lebanese districts. The median age was 28 (20-65; IQR: 8). Most of the participants held a Bachelor's degree (236, 76.4%), graduated from the Lebanese International University (LIU) (152, 49.2%) and worked full time (122, 39.5%) with an experience between 0 and 4 years in community settings (141, 45.6%).

Antibiotic prescribing practices varied, with 151 (48.9%) pharmacists prescribing them sometimes based on the symptoms. The most common antibiotic dispensed in general for pediatrics in the pharmacies was amoxicillin-clavulanate suspension (306, 99%), with pharyngitis/tonsillitis (303, 98.1%) being the most common case of pediatric infections encountered.

Only 38.5% of the pharmacists always checked an antibiotic's prescription for dosing errors and of the ones who noticed a wrong antibiotic's dose 284 (91.9%) contacted the doctor. When choosing self-correction, most pharmacists (291, 94.2%) referred to the leaflet.

Notably, to get information about the proper dosage, Medscape was chosen as the mostly used source (249, 80.6%).

Table 1, present at the end of the manuscript, shows the socio-demographic characteristics of the study participants and details related to antibiotics dispensing.

**Table 1:** Socio-demographic characteristics and details related to antibiotics dispensing.

Characteristics	Frequency (%)
Age (years)	28 (20–65)
Gender	
Male	153 (49.5)
Female	156 (50.5)
Marital status	
Single	210 (68.0)
Married	97 (31.4)
Widowed	1 (0.3)
Divorced	1 (0.3)
Level of education	
Bachelor of pharmacy	236 (76.4)
Pharm-D	67 (21.7)
Master	6 (1.9)
Educational institution	
LIU	152 (49.2)
LAU	20 (6.5)
BAU	25 (8.1)
LU	74 (23.8)
USJ	30 (9.7)
Abroad	8 (2.6)
Region	
Akkar	26 (8.4)
Baalback/hermel	6 (1.9)
Beirut	119 (38.5)
Bekaa	3 (1)
Kesserwan	28 (9.1)
Mount Lebanon	101 (32.7)
Nabatieh	1 (0.3)
North	16 (5.2)
South	9 (2.9)
Years of work experience in community pharmacy	
0–4	141 (45.6)
5–15	135 (43.7)
>15	33 (10.7)
Job position	
Owner	80 (25.9)
Full time	147 (47.6)
Part time	69 (22.3)
Pharmacy manager	13 (4.2)
Working hours per Day	
0–7	122 (39.5)
8	108 (35)
>8	78 (25.2)
Monthly income	
1,500,000–3,000,000 LBP	27 (8.7)
>3,000,001–5,000,000 LBP	77 (24.9)
>5,000,001 LBP	205 (66.3)
Sales incentives from the pharmacy for antibiotics dispensing	
No	271 (87.7)
Yes	38 (12.3)
Number of pediatrics patients encountered per day	11.45 (1–30)
What are the most common cases of pediatrics infections that you encounter in your pharmacy?	
Pharyngitis/Tonsilitis	303 (98.1)
Otitis Media	238 (77)
Urinary Tract Infection	96 (31.1)
Sinusitis	84 (27.2)
Gastroenteritis	185 (59.9)
Bronchitis/Bronchiolitis	212 (68.6)
Others	27 (8.7)
Number of oral antibiotics dispensed per day for children presenting with a prescription	4.39 (1–20)
Number of oral antibiotics dispensed per day for children without a prescription	2.61 (0–15)
Do you usually prescribe antibiotics according to the patient's symptoms?	
Sometimes	151 (48.9)
Always	22 (7.1)
Never	136 (44)
If yes what are the most common antibiotics dispensed in general for pediatrics in your pharmacy?	
Amoxicillin-clavulanate suspension	306 (99)
Trimethoprim-sulfamethoxazole suspension	101 (32.7)
Cefdinir suspension	178 (57.6)
Cefixime suspension	194 (62.5)
Cefuroxime suspension	111 (35.9)
Azythromycin suspension	180 (58.3)
Others	154 (49.8)
When you receive an antibiotic's prescription do you check if it contains any wrong dose?	
Never	2 (0.6)
Always	119 (38.5)
Sometimes	188 (60.8)
If you noticed a wrong antibiotic's dose what do you do?	
I don't do anything	1 (0.3)
Contact the doctor	284 (91.9)
I correct the dose because I am confident with my information	24 (7.8)
If you choose to correct the dose what do you usually do?	
I look at the leaflet	291 (94.2)
I calculate the new dose by a formula that I remember from the guideline	82 (26.5)
I check the correct dose from Google	132 (42.7)
Which sources do you use to get information about the appropriate dosing in pediatrics?	
American academy of pediatrics (AAP)	135 (43.7)
Food and Drug Administration (FDA)	35 (11)
Medscape	249 (80.6)
Labels on product containers	231 (74.8)
Physicians/pharmacists	49 (15.9)
Journals	237 (76.7)
Dosing applications	83 (26.9)
Drug information center (DIC) of the OPL	4 (1.3)
Others	37 (12)

PharmD, Doctor of Pharmacy; LIU, Lebanese International University; LAU, Lebanese American University; BAU, Beirut Arab University; LU, Lebanese University; USJ, Université Saint Joseph, LBP, Lebanese Pound; OPL, Order of Pharmacists of Lebanon.

### 3.2 Cases solutions about appropriate dosing of the most commonly encountered antibiotics among pediatrics

For the case scenarios, 303 participants (98.1%) correctly answered the first case (Amoxicillin-clavulanate in acute otitis media), while 232 participants (75.1%) correctly answered the second one (Trimethoprim-sulfamethoxazole in urinary tract infection). Case 3 which was about Azithromycin in acute bacterial pharyngitis and case 4 which was about Cefdinir for bacterial sinusitis, were correctly answered by 260 (84.1%) and 275 participants (89%) respectively. All these findings are presented in Table 2.

**Table 2:** Cases solutions about appropriate dosing of the most commonly encountered antibiotics among pediatrics (*n* = 309).

Cases	Frequency (%)
Case 1: Amoxicillin-clavulanate in acute otitis media	
Correct	303 (98.1)
Incorrect	6 (1.9)
Case 2: Trimethoprim-sulfamethoxazole in urinary tract infection	
Correct	232 (75.1)
Incorrect	77 (24.9)
Case 3: Azithromycin in acute bacterial pharyngitis	
Correct	260 (84.1)
Incorrect	49 (15.9)
Case 4: Cefdinir for bacterial sinusitis	
Correct	275 (89)
Incorrect	34 (11)

**Figure 1:**
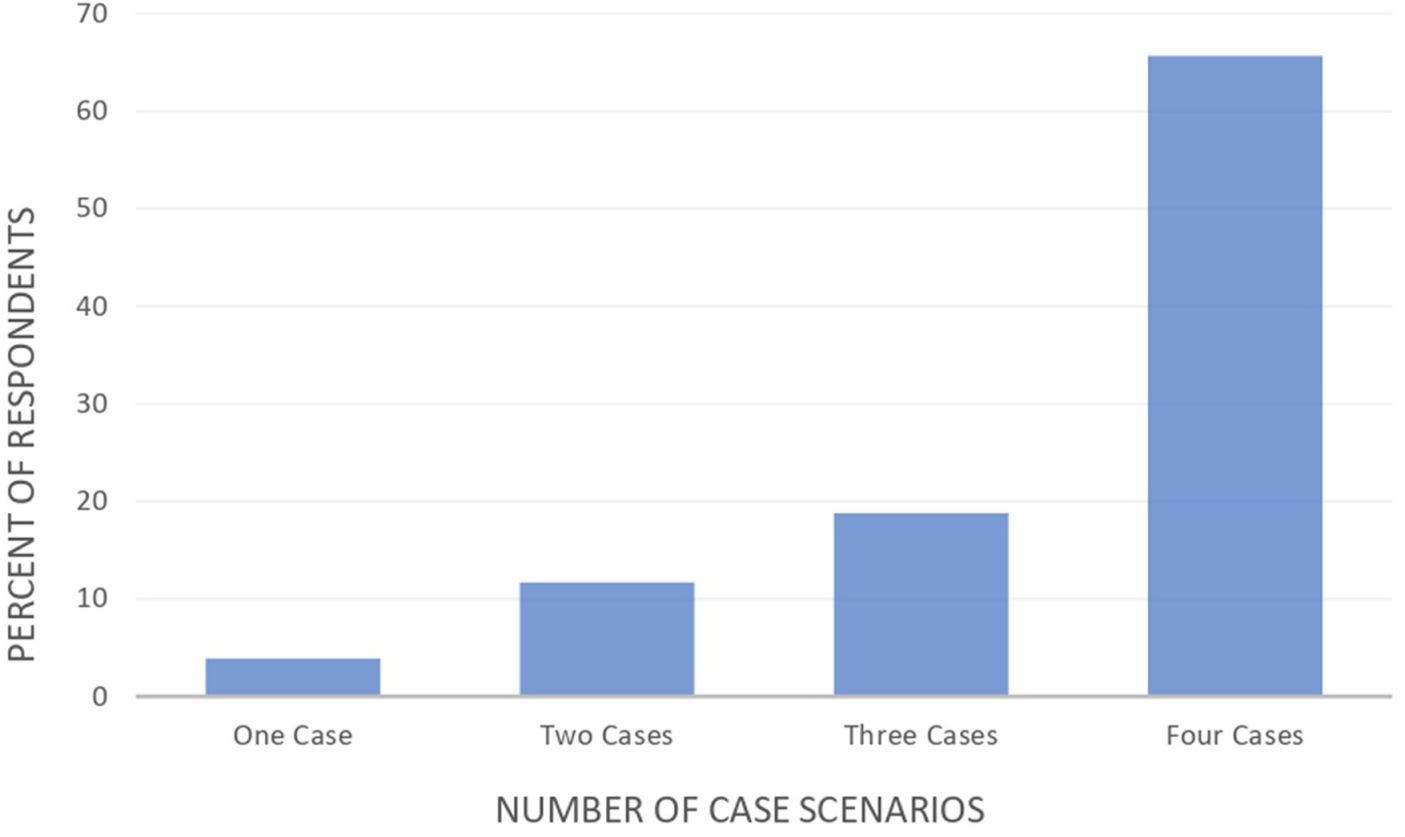
Number of correct answers of antibiotics’ cases.

The number of correct answers of antibiotics' cases are illustrated in Figure 1, whereby 65.7% of the participants answered the four cases correctly, 18.8% answered three cases correctly, 11.7% answered two and 3.9% answered one case correctly out of four.

### 3.3 Multivariate analysis for knowledge

The variables of age, marital status, educational institution, region, monthly income, years of experience in community pharmacy, gender, and working hours per day were included in the multivariate logistic regression analysis. As shown in Table 3, a monthly income of more than 5,000,001 Lebanese Pounds (LBP) (OR = 4.258, 95% CI: 3.135–12.434) and working more than 8 h per day (OR = 0.325, 95% CI: 0.112–0.938) were significantly associated with knowledge. Pharmacists working more than 8 h per day had significantly lower odds of adequate knowledge compared to those working fewer hours. Although pharmacists working exactly 8 h per day had higher odds than those working more than 8 h, this specific difference was not statistically significant in the model (*P* = 0.151).

**Table 3:** Bivariate and multivariate analysis testing the association of the demographic characteristics with the knowledge score and the factors affecting community pharmacists’ knowledge about dosing of antibiotics.

Demographic characteristics *N* = 309		Non knowledgeable *n* = 49 (15.9) No. (%)	Knowledgeable *n* = 260 (84.1) No. (%)	*P*-value	Multivariate analysis aOR (95% CI)	*P*-value
Gender	Male	18 (11.8)	135 (88.2)	0.051	Reference	
	Female	31 (19.9)	125 (80.1)		0.685 (0.325–1.442)	0.319
Age	Mean ± SD	49 28.69 ± 7.484	260 31.43 ± 8.257	0.032*	0.971 (0.869–1.084)	0.599
Marital Status	Single	40 (18.9)	172 (81.1)	0.032*	Reference	
	Married	9 (9.2)	88 (90.7)		1.814 (0.617–5.330)	0.279
Level of Education	Bachelor of pharmacy	39 (16.5)	197 (83.5)	0.377		
	Pharm-D	9 (13.5)	58 (86.6)			
	Master	1 (16.7)	3 (83.3)			
Educational institution	LIU	37 (24.3)	115 (75.7)	0.002*	Reference	
	LAU	1 (5)	19 (95)		6.621 (0.696–63.014)	0.1
	BAU	3 (12)	22 (88)		1.543 (0.377–6.321)	0.546
	LU	5 (6.8)	69 (93.2)		2.908 (0.896–9.434)	0.075
	USJ	1 (3.3)	29 (96.7)		6.255 (0.617–13.398)	0.121
	Abroad	2 (25)	6 (75)		1.507 (0.200–11.334)	0.691
Region	Akkar	8 (30.8)	18 (69.2)	0.03*	Reference	
	Baalback/Hermel	1 (16.7)	5 (83.3)		0.872 (0.068–11.129)	0.916
	Beirut	15 (12.6)	5 (87.4)		2.416 (0.733–7.967)	0.147
	Bekaa	2 (66.7)	1 (33.3)		0.403 (0.029–5.600)	0.498
	Kesserwan	4 (14.3)	24 (85.7)		1.388 (0.264–7.310)	0.699
	Mount Lebanon	14 (13.9)	87 (89.1)		2.488 (0.710–8.715)	0.154
	Nabatieh	1 (100)	0 (0)		0.22 (0.001–3.882)	0.323
	North	3 (18.8)	13 (81.3)		1.603 (0.288–8.941)	0.59
	South	1 (6.1)	8 (88.9)		3.488 (0.293–11.525)	0.323
Job Position	Owner	7 (88.8)	73 (91.3)	0.12		
	Full time	26 (17.7)	121 (82.3)			
	Part time	15 (21.7)	54 (78.3)			
	Pharmacy manager	1 (7.7)	12 (92.3)			
Monthly Income	1,500,000–3,000,000 LBP	12 (44.4)	15 (55.6)	0.0001*	Reference	
	>3,000,001–5,000,000 LBP	21 (27.3)	56 (72.7)		2.81 (0.923–8.557)	0.069
	>5,000,001 LBP	16 (7.8)	189 (92.2)		4.258 (3.135–12.434)	0*
Years of work experience in community pharmacy	0–4	31 (22)	110 (78)	0.025*	Reference	
	5–15	14 (10.4)	121 (89.6)		1.085 (0.434–2.712)	0.861
	>15	4 (12.1)	29 (87.9)		0.365 (0.027–4.871)	0.446
Working Hours per day	0–7	16 (13.1)	106 (86.9)	0.553	Reference	
	8	19 (17.6)	89 (82.4)		0.515 (0.208–1.273)	0.151
	>8	14 (15.9)	259 (82.1)		0.325 (0.112–0.938)	0.038*

PharmD, Doctor of Pharmacy; aOR, adjusted odds ratio; CI, confidence interval; LIU, Lebanese International University; LAU, Lebanese American University; BAU, Beirut Arab University; LU, Lebanese University; USJ, Université Saint Joseph, LBP, Lebanese Pound.

*Statistically significant values.

### 3.4 Factors affecting antibiotic dosing in pediatrics

**Figure 2:**
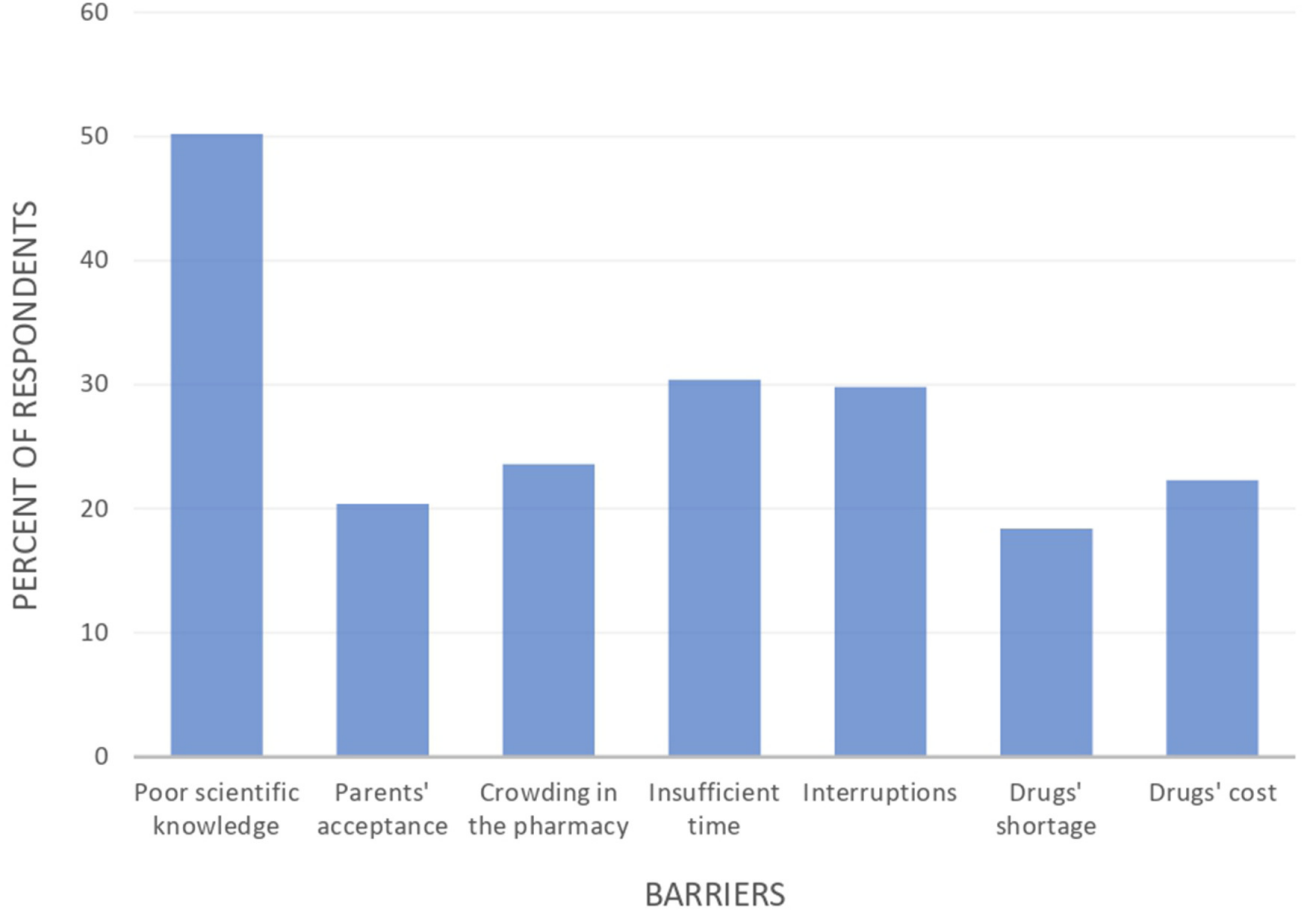
Barriers to inappropriate dosing of antibiotics.

Several obstacles were found in our study of community pharmacists' practices regarding the dosage of antibiotics in the pediatric population. Lack of sufficient scientific information regarding pediatric antibiotic dosing was the most frequently reported barrier by pharmacists. In contrast, drug shortages were the least frequently identified barrier. Figure 2 shows the barriers that may lead to inappropriate dosing of antibiotics.

## 4 Discussion

Antibiotics are the most incorrectly dosed classes of drugs in pediatrics and it is essential that pharmacists select the suitable and accurate antibiotics dose to significantly improve patient outcomes and reduce errors (5, 8, 15). The current study showed that 84.1% had adequate knowledge about antibiotic dosing in pediatrics, with a strong association between knowledge and both income and working hours.

A higher monthly income among community pharmacists was associated with more knowledge. This link is explained by the fact that a high income will inspire and urge community pharmacists to continually update their knowledge, enabling them to pay for the costs of educational or training courses (19). This is in line with the findings of Keewan et al. (14) who found that community pharmacists' knowledge of antibiotics was significantly correlated with monthly income as the only factor.

Lebanese community pharmacists who worked less than 8 h had higher knowledge scores than those who worked more than 8 h. Rahme et al. (20) reported that Lebanese community pharmacists encounter significant levels of fatigue related to their job which affects the pharmacist's professional practice, leading to prescription errors and adversely impacting patient safety. Studies by Lee et al. (21) and Johnson et al. (22) showed that long hours and irregular schedules can lead to chronic health problems, acute symptoms, and adverse health behaviors, which can affect the knowledge and productivity of pharmacists.

By providing four case situations, the current study's community pharmacists' knowledge level was evaluated. In contrast to a comparable Jordanian study where only 29% of community pharmacists calculated the correct dose of amoxicillin-clavulanate for acute otitis media, 98% of community pharmacists did so in the current study. The widespread use of amoxicillin-clavulanate in pediatrics in Lebanon may be due to community pharmacists' familiarity with the drug. In a study by Zahreddine et al. (11), amoxicillin-clavulanate was the most frequently used antibiotic for pharyngitis (47.2%) and otalgia (70%) in pediatrics.

Almost three quarters of the pharmacists provided the correct response for trimethoprim-sulfamethoxazole for urinary tract infection, which was prescribed the least at pharmacies (32.7%). This may reflect limited familiarity with its pediatric dosing due to infrequent use. Clinically, TMP-SMX dosing in children can be particularly challenging because it is weight-based and requires careful adjustment to avoid sub-therapeutic levels or toxicity. This complexity likely contributes to the lower accuracy in dosing observed across studies. However, a study from the Netherlands (23) showed that it was prescribed to children in sub-therapeutic doses, and a study from Jordan (14) showed that only 16% of community pharmacists correctly determined the dosage of the drug.

Azithromycin in acute bacterial pharyngitis had 84.1% correct answers. By comparing our findings to those of the Keewan et al. (14) study, we found that 56% of community pharmacists calculated the azithromycin dose accurately in cases of acute bacterial pharyngitis. Furthermore, the Haddadin et al. (24) investigation revealed that 57% of azithromycin cases received doses that were greater than advised. Furthermore, according to research by Brown (25) and Keewan (14) et al., only 41% and 36% of community pharmacists correctly estimated the dosage of cefdinir for acute bacterial sinusitis.

This disparity in percentages indicates that Lebanese community pharmacists are the primary healthcare providers in the Lebanese community; and the variation between research may be influenced by different study objectives, designs, populations (age groups, sample size) and pharmacy curriculum between the Lebanese and the Jordanian schools of pharmacy. Curriculum gaps between Lebanese and Jordanian pharmacy programs may contribute to disparities in knowledge and practice. Lebanese students frequently rely on international resources like Medscape due to the absence of standardized local dosing charts, while Jordanian programs provide more structured clinical curriculum and standardized tools. This highlights the need for curriculum reform and national guideline development in Lebanon.

Community pharmacists can use different resources to determine the correct antibiotic doses. Medscape is the most preferred resource by most pharmacists as it is free, simple, reliable and provides unlimited access to the whole network of websites (26). In a previous study conducted in Senegal (27), pharmacists relied on the internet (65.9%), their colleagues (63.7%), and books (53.8%), while Keewan et al. (14) found that medication leaflets, training, and university courses (respectively 52.1%, 44%, and 36.5%) were the resources used most frequently by pharmacists. Furthermore, according to Brown et al. (25), community pharmacists also used other resources such as the internet, the Micromedex database, facts and comparison, and Lexicomp.

The inappropriate dosage of antibiotics in pediatrics has been linked to a number of barriers. According to the study, poor scientific knowledge was identified as the primary factor contributing to dose errors by 50.2% of pharmacists. This was also observed in previous studies conducted by Keewan et al. (14) and Zahreddine et al. (11) in Jordan and Lebanon. Additionally, 86.1% of pharmacists in Zahreddine et al. (9) study reported that socio-economic problems contributed to antibiotic misuse. In the current study, 22.3% and 18.4% of pharmacists reported that the cost and lack of medications, respectively, had an impact on antibiotic dosing. The barrier of parental acceptance was also observed in both studies. The barrier of parental acceptance was also observed in both our study and the study of Zahreddine et al. (11).

The findings of this study have important implications for continuing education, national guidelines, and policy development. Identifying knowledge gaps can guide the design of targeted educational programs to strengthen professional competencies and improve practice. These results can also support the revision of national guidelines by providing locally relevant evidence. Moreover, highlighting factors such as income and working hours emphasizes the need for policy interventions that promote equitable access to training and address systemic barriers to professional development.

Although this study was the first to investigate the knowledge of community pharmacists regarding antibiotics dosing in pediatrics, there are a few limitations on this study, which was based on an electronic survey. First of all, when evaluating the results, it is important to note that the validity and reliability (such as Cronbach's alpha), were not evaluated in this study. Second, the cross-sectional form of the study makes it challenging to pinpoint the temporal relationship between exposure and results. Third, the current findings may not be generalizable due to a potential risk of selection bias connected with the snowball sampling technique. Nonetheless, it is thought that this risk is reduced because pharmacists from all around Lebanon were included in our sample. Fourth, the web-based survey is one of the most effective ways to quickly gather data, but it also carries the danger of selection bias because it might have left out senior pharmacists who are less good with technology. Nevertheless, as a result of self-reporting evaluation techniques, recall bias could still develop. Nonetheless, it is thought that this bias is reduced because the survey used an English language that all Lebanese pharmacists are familiar with.

## 5 Conclusion

Our findings showed that the majority of pharmacists are knowledgeable about the proper antibiotic dosage for pediatric patients. Notably, income and working hours were significantly associated with knowledge levels, suggesting that these socioeconomic factors may influence professional performance. To improve pharmacy practice and avoid dosing errors, several barriers still need to be tackled. Addressing the economic challenges facing pharmacists and the healthcare system in Lebanon is crucial to ensure that pharmacists can continue to provide high-quality care to their patients and maintain their professional development. This may include advocating for fair compensation for pharmacists, inaugurating more effective platforms and targeted educational programs, developing policies to support the sustainability of the healthcare system in Lebanon; and thus guarantee a good practice in the community.

## Data Availability

The original contributions presented in the study are included in the article/Supplementary Material, further inquiries can be directed to the corresponding author.
